# Changes in Alpine Butterfly Communities during the Last 40 Years

**DOI:** 10.3390/insects13010043

**Published:** 2021-12-30

**Authors:** Simona Bonelli, Cristiana Cerrato, Francesca Barbero, Maria Virginia Boiani, Giorgio Buffa, Luca Pietro Casacci, Lorenzo Fracastoro, Antonello Provenzale, Enrico Rivella, Michele Zaccagno, Emilio Balletto

**Affiliations:** 1 Department of Life Science and Systems Biology Turin University, 10123 Turin, Italy; simona.bonelli@unito.it (S.B.); cri.entessa@virgilio.it (C.C.); giorgio.buffa@unito.it (G.B.); luca.casacci@unito.it (L.P.C.); lorenzo.fracastoro@gmail.com (L.F.); michele.zaccagno@unito.it (M.Z.); emilio.balletto@unito.it (E.B.); 2 Gran Paradiso National Park, 10135 Turin, Italy; 3 Institute of Geosciences and Earth Resources, Italian National Research Council, 56124 Pisa, Italy; mariavirginia.boiani@igg.cnr.it (M.V.B.); antonello.provenzale@cnr.it (A.P.); 4 Regional Agency for Environmental Protection, ARPA, 10135 Turin, Italy; enrico.rivella@arpa.piemonte.it

**Keywords:** mountain ecosystem, maritime alps, community ecology, climate change, reforestation, geomorphic disturbance, long-term changes

## Abstract

**Simple Summary:**

The Alps are among the most vulnerable ecosystems to climate change, as these modifications take place at a faster pace at higher elevations. Since butterflies are ideal model systems to investigate species responses to climate and habitat changes, we monitored a butterfly community of the Valasco valley in the Maritime Alps (NW Italy) by three sampling events covering a period longer than 40 years. We observed an overall increase in mobile, tolerant and thermophilous species, which eventually might cause an overall loss of community distinctiveness. The variations observed in the butterfly community can be explained by the notable increase in maximum temperatures and the reduction of grasslands, along with the increase of woodlands, supporting the hypothesis that local warming and land-use changes have ultimately affected the butterfly community composition.

**Abstract:**

Our work aims to assess how butterfly communities in the Italian Maritime Alps changed over the past 40 years, in parallel with altitudinal shifts occurring in plant communities. In 2019, we sampled butterflies at 7 grassland sites, between 1300–1900 m, previously investigated in 2009 and 1978, by semi-quantitative linear transects. Fine-scale temperature and precipitation data elaborated by optimal interpolation techniques were used to quantify climate changes. The changes in the vegetation cover and main habitat alterations were assessed by inspection of aerial photographs (1978–2018/1978–2006–2015). The vegetation structure showed a marked decrease of grassland habitats and an increase of woods (1978–2009). Plant physiognomy has remained stable in recent years (2009–2019) with some local exceptions due to geomorphic disturbance. We observed butterfly ‘species substitution’ indicating a general loss in the more specialised and a general gain in more tolerant elements. We did not observe any decrease in species richness, but rather a change in guild compositions, with (i) an overall increased abundance in some widespread and common lowland species and (ii) the disappearance (or strong decrease) of some alpine (high elevation) species, so that ‘resilience’ could be just delusive. Changes in butterfly community composition were consistent with predicted impacts of local warming.

## 1. Introduction

Climate studies strongly suggest that atmospheric alteration is already occurring and that changes in atmospheric composition are altering weather and climate processes. Warming of the climate system is unequivocal, as is evident from many observations of an increase in global average surface air temperatures [1]. In mountain regions, the warming rate (0.3 °C per decade, with a likely range of ±0.2 °C, [2]) outpaces the global rate (0.2 ± 0.1 °C per decade, [2]). Indeed, when observations at lower and higher elevations are compared, alterations are generally enhanced above 500 m [3,4]. Closer investigations reveal that during the second half of the 20th century and the beginning of the 21st century the climate of the western Alps has been characterised by an increase in maximum temperatures of up to 2 °C, a more modest increase in minimum temperatures and little or no trend in precipitation data, also indicating that these changes are happening at a faster pace since the mid-1980s [5,6]. Given the central role of climate in governing the natural environment of mountain areas and the climate dependence of most biological processes in these harsh environments, high vulnerability of such systems under the impacts of a rapidly changing climate is to be expected [7]. Mountain ecosystems provide interesting and valuable models for the early detection of climate change and for investigating its indicators and impact on ecological systems [8,9,10]. The uniqueness of mountains in detecting climate-driven impacts is related to rapid changes in climate, vegetation and hydrology with respect to elevation, within relatively limited horizontal distances and to the vast biodiversity hosted by their complex landscape [11]. Although responses vary along with climate change rates, ecological conditions and biogeographical regions [12], global-scale effects of climate change on the biology of many plant and animal species in mountain ecosystems have already been observed [13]. Even if climate might be the main driver at a large spatial scale and its variations are easily modelled to tackle several general questions [14], smaller-scale factors can alter local species responses [15].

During the last decades, changes in the vegetation cover have been recorded in many mountain ecosystems. Past studies revealed changes in dominant plant species at a global scale [16], while locally they show an increase in the extent of tree and shrub coverage (since 1960, forest cover in the Italian Alps doubled its extension [17]; for differences between the Alps and Apennines see [18]; for the French Alps [19]; for the Swiss Alps [20]) and a general upwards altitudinal shift of the treeline ecotone (for the Swiss Alps [21,22]; for the southwestern French Alps [23]; for the Scandinavian Mountains [24]; for a global survey [25]), but site topography and small-scale factors might outweigh the effects of global warming ([26]; see the “conservative” pattern of the Central European Alps [27] and references therein). On the other hand, climate change is not the only disturbing factor; therefore, assessing a direct role of global warming on these modifications is difficult in the Alps and on the hilly slopes of southern Piedmont (Italy), where landscapes have been modelled by millennia of human pressure, recently followed by changes in land use as agricultural and pastoral intensification or abandonment, or land abandonment (for the European Alps see [28,29]; for the Italian Alps [30] and Piedmont [31,32]). European mountain ecosystems and Alpine faunas, in particular, are affected by changes in pastoralism [33] which, in the past, contributed to lower the tree line and halt vegetation encroachment by maintaining open grasslands [34]. Geomorphic disturbance is also well known for causing changes in the distribution patterns of plant communities, particularly at the subalpine and alpine levels and in the presence of steep slopes and periodic avalanches [35,36,37].

As a consequence, assessing the relative roles of geomorphic disturbance, land-use changes and global warming in shaping current ecological features is often challenging, as well as to hypothesise how these factors may interact in the near future [21,31,38,39]. Since climate is defined as «the statistical description in term of means and standard deviation of notable factors, over a long period, usually 30–50 years» (World Meteorological Organisation), assessing the impacts of climatic variation on biological systems will generally require that data records span over long periods, both as concerns climate variables and biological observations [40]. However, long-term series are rarely available (but see [41]).

Butterflies are ideal model systems to investigate species responses to climate and habitat changes [41,42,43,44]. Primarily, because individuals and populations are strongly influenced by their abiotic environment and ecological conditions [45,46], butterflies react more promptly than many other organisms to environmental change [47]. Since climate change can alter species dynamics, results obtained by the study of changing butterfly communities in a varying climatic scenario will provide a starting point for research on a wide variety of other herbivorous insects [48,49,50]. Butterflies, as well as biotic components in general, may adapt to changing environments showing phenological plasticity and/or genetic changes (see [51] for a review on invertebrates), as well as by shifting their spatial distribution moving towards favourable conditions [52]. Thus, as the climate warms, adults tend to emerge earlier [53,54], flight periods last longer [54] and numbers of generations in multivoltine species increase [55]. In mountain ecosystems, upwards shifts of species linked to colder conditions can be observed [8,52]. Thermophilous species could profit from global warming and extend their occurrence by spreading northwards or to higher elevations. However, species movements occur only if suitable and relatively uninterrupted habitats are available (mainly represented by open areas in the Alps), and often mirror range contractions [56,57,58]. In the worst cases, global warming and loss of suitable habitat drive local extinctions, but species responses might also lead to less severe changes at the community level [59]. In a long-time series study (1840–2013), Habel and colleagues [59] found an overall impoverishment of grassland butterfly communities in southern Germany, with a decrease in the proportion of specialists (already endangered species) and low-dispersal butterflies. Even considering a short time frame, Cerrato et al. [60] observed quite significant variations in butterfly assemblages of the NW Italian Alps which can be partially explained by both global warming and microhabitat changes. These authors found an overall increase of generalist and highly vagile species in five years, leading to an incipient community homogenisation [60].

Although, in the mountains, climate alterations can lead to rapid changes in ecosystems and in the way species occur in space and interact with each other in communities, long-term studies on variations of invertebrate assemblages are surprisingly rare (but see [60]) in the Alps. Therefore, in 1978, 2009 and 2019 we sampled butterfly species occurring in seven plots of the SW Italian Alps, located at elevations ranging from 1200 to 2200 m and representing different habitats, to document changes in butterfly community compositions that occurred in the past 40 years. Butterfly species are prone to fluctuate in individual numbers and our surveys were limited to three points in time (1978, 2009 and 2019), but the statistical approach, the spatial and temporal scale and the focus on species occurrence (instead of species abundance) that we chose, still allow us to get crucial insights on the effects of climate and vegetation changes on butterfly community compositions.

## 2. Materials and Methods

### 2.1. Study Area

Butterfly communities were sampled in the SW Italian Alps (Maritime Alps, Valasco Valley) at seven sites (hereafter “plots”), scattered along an altitudinal gradient (1300–1900 m) and covering different habitat types, representative of the natural variability of the mountain system under study (Table 1).

The study area is well-known for being crucial for the conservation of the Italian insect fauna and for its rich butterfly assemblage [61]. It falls within the Site of Community Importance (SCI: “Argentera” IT1110053) included in the Maritime Alps Natural Park and it is listed among the 32 Italian priority sites for conservation known as “Prime Butterfly Areas” [62]. The position of each plot was georeferenced in 1978 [63], allowing the repetition of the surveys in 2009 and 2019 (Figure S1). Ente di Gestione delle Aree Protette delle Alpi Marittime granted all permits necessary to perform our study within the Natural Park.

### 2.2. Climate Data

Historical climate data were obtained originally from the NWIOI dataset (North West Italy Optimal Interpolation—OI; [64,65,66]), processed by ARPA Piedmont (Regional Agency for the Environmental Protection), for which the ERA-40 re-analysis of the European Centre for Medium Range Weather Forecasts and raw meteorological station data were used as a background field. These data cover the period from 1958 to 2019 and include daily maximum and minimum temperature and daily cumulative precipitation. The NWIOI temperature and precipitation data were downscaled by linear interpolation to reach a resolution of 25 m, estimated to be a relevant scale for butterfly ecology. The interpolated temperature data were also corrected for elevation anomalies, using a monthly-averaged temperature lapse-rate [67]. The interpolated precipitation and elevation-corrected temperature data were finally aggregated to obtain monthly means, used in subsequent analyses. Trends and anomalies were evaluated by averaging data at annual and seasonal scales.

To assess possible altitudinal shifts of butterfly species, the climate data were used to identify two additional plots at higher elevations where vegetation and climatic conditions reflected those found at lower altitude sites in 1978. An altitudinal band representing the climatic conditions of the first year of sampling (1978) was obtained by averaging temperatures recorded in the preceding ten years (1968–1977). The same approach was used to identify the equivalent climatic altitude band for the last decade of sampling (2009–2018), generally at higher elevations. In this way, a new altitude band reflecting the temperature conditions found at the original altitude band of 1978 was identified, and two new additional plots were positioned in this higher-elevation band.

To analyse the time series of meteorological variables, we used two approaches. First, we adopted a Seasonal-Trend decomposition procedure based on the LOESS smoother (STL, see Cleveland et al. [68]). This method represents a filtering procedure for decomposing a time series into three components, i.e., seasonal—the periodic component present in our signal; trend—over the years; residuals—the remaining unexplained components and therefore the anomalies [68]. Second, we estimated long-term linear trends in deseasonalised temperature and precipitation time series and determined their significance using an F-test. We carried out these analyses for the full time series (1958–2019) and for two separate time periods (t1 = 1958–1979; t2 = 1980–2019), to explore possible changes in the trend intensities.

To describe the climatic characteristics of the three sampling years (1978, 2009 and 2019) quantitatively, we used variables known to provide indices of referenced limitations to butterfly growth and survival rates: (i) annual daily temperature sum above 5 °C degrees from January until February, April, June and August (GDD5; surrogate for the developmental threshold for butterfly larvae); (ii) mean temperature of the coldest month (MTCO; related to overwintering survival), as suggested by Hill et al. [69] and following literature [70,71]; (iii) mean annual temperature (T mean, °C) and (iv) mean temperature for each season (DJF, winter; MAM, spring; JJA, summer).

Statistical analyses were carried out on CDO 1.9.8 (Climate Data Operator, Max Planck Institute for Meteorology 2019).

### 2.3. Vegetation Data

Changes in land cover were assessed through the analysis of: i) the aerial photographs taken in 1978 (source IPLA archives, Piedmont) and ii) ortho-rectified aerial images from 2010 and 2018 (source Piedmont Region administration, freely available from https://www.geoportale.piemonte.it (accessed on 30 October 2019)). Pictures taken in 1978 were rectified on the basis of 10 ground control points retrieved from a recent (2012) orthoimage. Aerial images taken in 1978, 2010 and 2018, representative of the three periods of butterfly community sampling, were analysed for circles with a radius of 200 m around the centre of transects. Five types of land cover were identified: woodland, sparse woodland (i.e., low density of trees), scrubs, grasslands and screes. Each land cover type was quantified as a percentage of the total circle under analysis, with the exception of parts not visible in one or more images or occupied by the road. Field observations performed in 2009 and 2019, during the butterfly sampling period, were used to descriptively confirm patterns observed in the orthophotographs taken in 2010 and 2018. We compared percentages of land cover between the years and for each category by Friedman test and pairwise Wilcoxon signed-rank test. For land cover types that significantly differed between the years, we quantified the amount of change for each plot by applying Euclidean distances. First, we estimated changes for each land cover category and then for all the categories grouped, in order to obtain an aggregated measure of vegetation change per plot. To test the hypothesis that a greater change in vegetation cover determined a higher turnover in butterfly community composition, and to identify which land cover type was the most influential, we calculated Spearman rank correlation between Euclidean distances and Bray–Curtis distance matrices applied to butterfly community composition.

### 2.4. Butterfly Communities

Butterflies were sampled by linear transects [72], one per habitat type (plot—Figure S1), fixed in length (about 300 m) and time (45 min) in sunny days with scarce wind, between 10 am and 3 pm. All butterflies were caught by a 40 cm diameter net; only individuals hard to be identified in the field (e.g., some *Pyrgus* or lycaenid species) were sacrificed and identified in the laboratory. In 1978, the sampling period was limited to two sampling sessions between the end of July and the beginning of August, the ideal period to study butterflies in mountain ecosystems, because it represents the flight peak of mountain communities (Table S2). In 2009 and 2019, the sampling period was extended to cover the whole flight season from the beginning of June until the end of August, with 7 sampling sessions carried out every 7–10 days, depending on weather conditions (Table S2). The reason for lengthening our sampling time was to cover almost all the potential flight season, as to make sure that the ‘*no-more-found species*’ had really disappeared during our 40-year time frame and had not just suffered some phenological shift. The number of individuals per each species was counted for 2009 and 2019 sampling events only and used for short-time comparisons (see Section 2.4.2 and Section 3.3.2). Long-time analyses (see Section 2.4.2 and Section 3.3.1) are based on species occurrence; therefore, we could assess species loss or phenological shifts in a 40-year time frame but did not account for variation in species abundances.

#### 2.4.1. Ecological Classification of Species

Species were characterised by their ecological requirement, following Balletto and Kudrna [73] and considering their: (i) general habitat requirements (woodland, ecotonal, open herbaceous, screes); (ii) altitudinal preferences (generalist, low altitude, high altitude); (iii) stenophagy (monophagous, strictly oligophagous (one foodplant genus), oligophagous (one foodplant family)) (for the species list see Table S1).

#### 2.4.2. Changes in Butterfly Communities

Our data were gathered in three sampling events far away in time (1978, 2009 and 2019). In order to avoid any bias due to natural fluctuations in the number of individuals, we based our comparison primarily on species occurrence, and we assessed community composition variations. We analysed butterfly communities sampled at Valasco valley, focusing both on the long- (LTP—1978–2019) and the short-time period (STP—2009–2019). To test LTP changes in species richness, we used a subset of the sampling sessions carried out in 2009 and 2019, which were limited to those corresponding to the time frame monitored in 1978 (two sampling sessions: 30th July and 8th August for 2009, 30th July and 10th August for 2019). Short-term variations that occurred in the last ten years were assessed by comparing the data recorded in 2009 and 2019 for the whole flight season (from the beginning of June to the end of August). We compared changes in species richness, number of plots occupied by each species and the proportion of species and of occupied plots for each ecological category among years. These analyses were carried out using ANOVA/Friedman test for LTP, and Student *t*-test/Wilcoxon signed-ranked test for STP according to data distribution. Shapiro–Wilk tests were used to assess data distribution. All analyses were performed using paired tests; *p* values were adjusted according to Benjamini–Hochberg corrections. For the STP, we assessed the variation of Shannon indexes calculated for each plot between 2009 and 2019 by applying the Student *t*-test for paired samples.

For the 2009 and 2019 datasets, we applied a recently developed rarity index, *rr* index [74] to evaluate species composition differences by detecting possible rarefaction of certain species. This index is applied to the whole study area and, following Rabinowitz’s [75] scheme, it evaluates the geographical range of each sampled species by the coordinates of the observation (gri), the maximum number of the species habitat (hsi) and the maximum population size sampled (psi); the final *rr* index is the average of all these indices. Therefore, it functions as a local index and the classification as “rare” for a species must be intended only for the area at which it is applied (i.e., it does not mean that the species is globally or regionally rare, but it is rare in the sampled community). All of these measures range from 0 to 1, the closer the index value gets to 0, the more common the species is, while the closer it gets to 1, the rarer is the species. Following Maciel [74], rare species have an *rr* index higher than the average *rr* index calculated for the whole community. Differences in the proportion of rare species between the butterfly communities sampled in 2009 and in 2019 were tested by Wilcoxon tests.

We analysed community compositions by testing for changes in location (significant changes in community composition per plot over time) and dispersion over the years (significant changes in observed differences in community composition among plots, over time). Changes in location were tested by applying non-parametric MANOVA to Bray–Curtis distance matrices, to test if the multivariate centroids of species composition were, or were not, similar in the three groups [76,77]. Non-parametric MANOVA was performed by the function *adonis* of the *vegan* package [78]. The significance of the test was assessed by using F-tests based on sequential sums of squares obtained from permutations of the raw data (999 permutations). To account for the temporal structure and the spatial dependencies of our sampling design (7 plots at 3 points in time), we applied restricted randomisation, which did not allow for permutations across samples. Changes in dispersion were tested by the *betadisper* function of the *vegan* package, a multivariate analogous of the Levene’s test for comparing group variances [76]. Non-Euclidean distances between objects and group centroids were handled by reducing the original distances to principal coordinates. To test for significance, we applied a similar randomisation approach, as previously explained.

To test for species responses over time, we used the ‘indicator species’ analysis (IndVal method) proposed by Dufrêne and Legendre [79]. The IndVal method combines the “specificity” of a species (its uniqueness in one of two years) and its “fidelity” (its frequency within one year). For each species, IndVal may range from 0 (no indication) to 1 (maximum indication). Statistical significance of IndVal was tested by using a Monte Carlo test, based on 999 randomisations, and performed by *indicspecies* package [80]. Indicator Species Analysis (IndVal method) allowed us to identify species that:

- were common in all time frames (highest IndVal values in the 1978 + 2009 + 2019 combination);
- abruptly increased their presence during the last decade (highest IndVal in 2009 or 2019 or 2009 + 2019);
- were common in 1978, but strongly suffered during time (highest IndVal in 1978).

To assess which species had completely disappeared over time descriptively, we used all data from the sampling sessions performed in 2009 and 2019 and compared them with data collected in 1978.

#### 2.4.3. Community Temperature Index

We used the “Species Temperature Index—STI”, to relate community composition to the species climatic niches [81]. The data were obtained by the updated version of the database on Lepidoptera Papilionoidea [82] build up for the project ‘CkMap’ by the Ministry of the Environment, Land and Sea [83]. Nowadays, this dataset includes over 388,000 individual records (the 2007 version included 60,000 records) mapped on 10 × 10 UTM grids. Temperature data were obtained by the maps of Metz et al. [84], using annual mean temperature (BIO1). The “Community Temperature Index—CTI” was calculated by averaging STI for all the species counted in each plot, for each year (see Cerrato et al. [60] for a detailed description of the procedure). CTI was used to evaluate species changes over time by applying an ANOVA/Friedman test for paired samples.

Where not otherwise specified, results have been reported in the main text as means ± standard errors. All the statistical analyses were carried out on R 3.6.1 (R Core Team 2019).

## 3. Results

### 3.1. Climate Data

**Figure 1:**
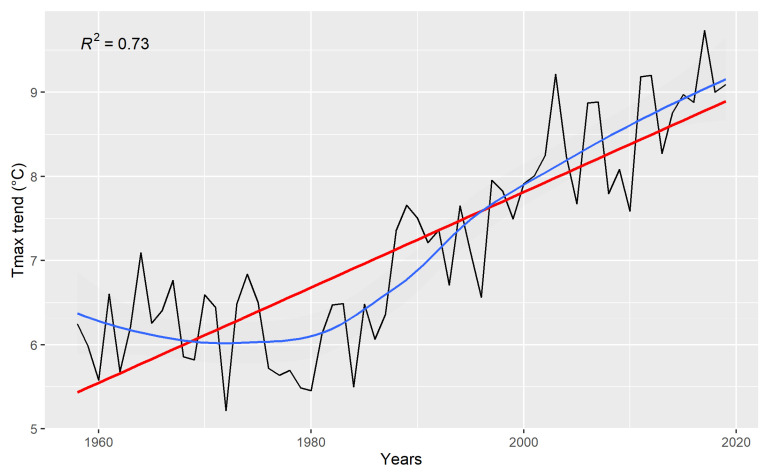
Maximum temperature deseasonalised trend values as a function of time, for the whole time period 1958–2019; on the x axis the years; on the y axis annual temperature values (°C). In red the linear trend through time, in blue the local regression (LOESS). R^2^ is referred to the linear trends.

Maximum temperatures significantly increased throughout the analysed time period (R^2^ = 0.73, F = 159, df = 1, *p* < 0.001), with a positive linear trend of +0.05 °C/year. The strongest increase was observed for the second time period (1980–2019: +0.08 °C per year, R^2^ = 0.78, F = 140.1, df = 1, 38, *p* < 0.001; Figure 1) while maximum temperatures did not increase between 1958 and 1979 (R^2^ = 0.04, F = 0.95, df = 1, *p =* 0.34).

No significant trend was observed for minimum temperatures in the NWIOI data set for the study area, either for the whole time series (R^2^ = 0.01, F = 1.96, df = 1, *p =* 0.16) or for the series divided in the two time periods (t1, R^2^ = 0.01, F = 1.23, df = 1, *p =* 0.28; t2, R^2^ = 0.06, F = 3.52, df = 1, *p =* 0.06). In the first years, however, minimum temperatures were usually measured at a fixed time during the night and could reflect the true minimum daily temperature only approximately. For precipitation, no trend was observed for the total period (R^2^ = 0.003, F = 0.23, df = 1, *p* = 0.63) or for the first subset (t1 1958–1979, R^2^ = 0.04, F = 0.88, df = 1, *p* = 0.35), in keeping with the results of Ciccarelli et al. [5]. Slight evidence of a precipitation increase was highlighted in the second period (1980–2019, R^2^ = 0.24, F = 13.69, df = 1, *p* < 0.0001) but the R^2^ value is too low to identify a real increasing trend. Comparing climatic variables between 1978, 2009 and 2019, we observed that 2009 and 2019 were hotter than 1978, but not different from each other in terms of annual mean temperatures. At a seasonal level, 2009 was a peculiar year with very low temperatures in winter, even lower than those in 1978 and much higher temperatures in spring and summer, while 2019 had a mild winter, a colder spring and a much hotter summer (Table 2).

### 3.2. Vegetational Data

**Figure 2:**
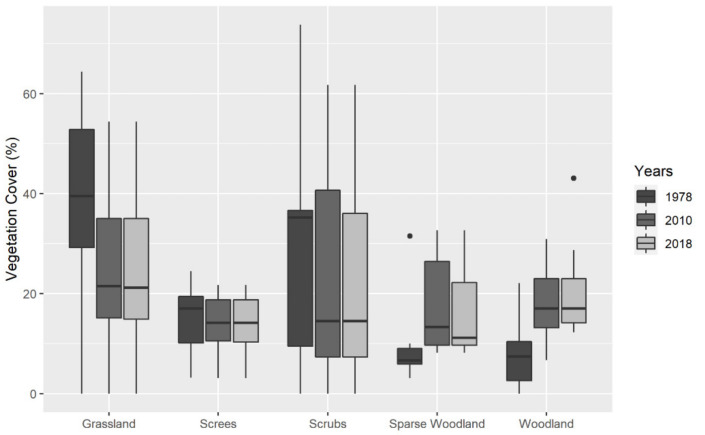
Percentage of land cover types for each plot in 1978 (dull grey), 2010 (mild grey) and 2018 (light grey) represented with boxplots. Boxplots show median, quartile, maximum, and minimum values; outliers are indicated by black dots.

The comparison of the adapted aerial photographs (1978) and the orthophotographs (2010 and 2018) highlighted that significant changes in land cover occurred over time (Figure 2).

Over the period 1978–2018 we observed an impact of small wood cuttings and avalanche damage to the woodland cover, but we also recorded a significant increase in tree cover (Friedman χ^2^ = 7, df = 2, *p* = 0.030). Between 1978 and 2010, the mean wooded surface doubled (Wilcoxon_1978–2010_ z = 1, df = 2, *p* = 0.047), while changes between 2010 and 2018 were negligible. Fully wooded + sparsely wooded surfaces rose from 18.1 ± 4.1% to 37.7 ± 5.9%. The only observed exception was at site rh2, strongly affected by geomorphic disturbance, where the total wood cover decreased by 5.7% in the long-term period (1978–2018). Expanding trees were mainly represented by beech and larch at low elevations and by larch in all other sites.

An increase in the individual sizes of previously existing trees, generally determining the closure of the canopy layer, was accompanied by the presence of new tree clusters in grasslands and heathlands. In parallel to the observed increase in tree coverage, we also noticed a significant decrease (Friedman χ^2^ = 11.2, df = 2, *p* = 0.003) in the areas occupied by grassland (1978–2010: −13.1 ± 0.1%; 1978–2018: −13.2 ± 0.1%). Scrubs also slightly decreased since 1978 (Friedman χ^2^ = 4.8, df = 2, *p* = 0.09; Figure 2). During the last decade (2010–2018), no significant differences were assessed in the vegetation cover, the main change being linked to the density of trees, with an increase of fully wooded surfaces and a decrease of the sparsely wooded areas, whereas the sum of the two changed only slightly (+1.5%). We observed a positive and high correlation only between changes in wooded areas (Euclidean distances) and changes in butterfly communities (Bray–Curtis distances) (ρ = 0.785, *p* = 0.048), indicating that the expansion of wood cover enhanced dissimilarities between butterfly assemblages. No significant correlations were found between butterfly communities and other land cover types (grassland, scrubs and screes).

### 3.3. Changes in Butterfly Communities

**Figure 3:**
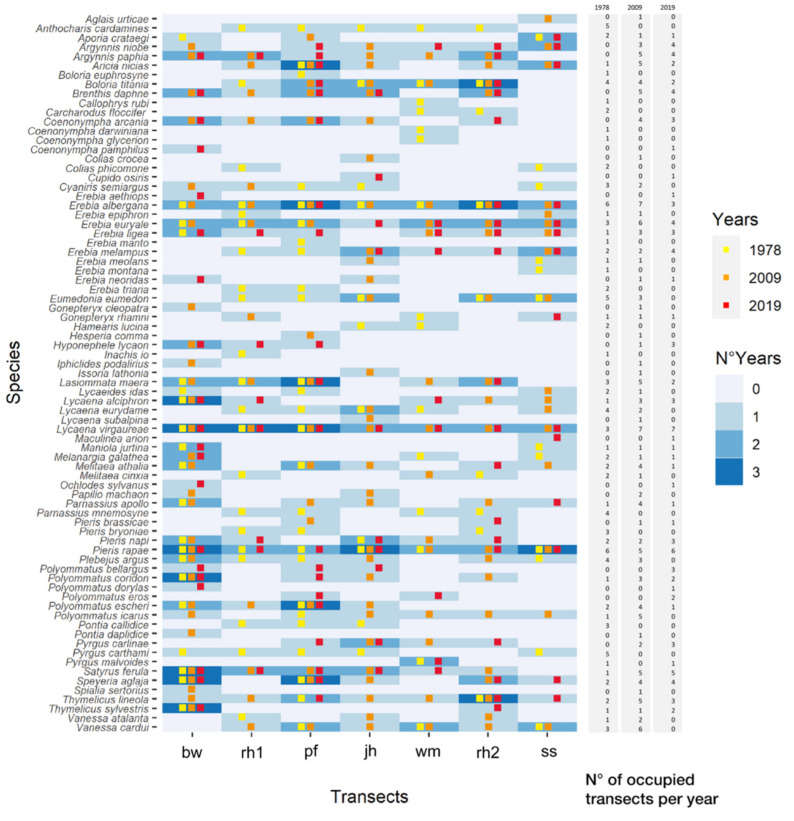
Graphical representation of all species collected during the last week of July and the first week of August in each plot for each year. Background colours, from light blue to blue, indicate the number of years in which the species was present. Yellow squares mean that the species was observed in 1978, orange squares in 2009 and red in 2019. The number of occupied plots per species in each year is reported in the three rightmost columns.

The whole butterfly community recorded in the three years (considering only the two central sampling events) encompasses 77 species. In detail, 51 species were recorded in 1978 (16 exclusive of the period), 52 species in 2009 (10 exclusive) and 40 species in 2019 (8 exclusive). Species common to all years were 23 (Figure 3, Table S1). Considering the whole sampling season (June-August), in 2009 and 2019 we recorded a total of 85 species, 55 of which were present in the two years, while 20 were only reported for 2009 and 10 for 2019.

#### 3.3.1. Long-Time Series Analysis (1978–2019)

Mean species richness per plot was slightly higher in 2009 (S_1978_ = 16.14 ± 2.39; e_009_ = 20.43 ± 2.51; S_2019_ = 13.86 ± 2.05), but differences across the three sampling events were not significant (ANOVA F = 2.272, df = 2, *p* = 0.146). The mean number of occupied plots per species (N_1978_ = 1.47 ± 0.18, N_2009_ = 1.86 ± 0.23, N_2019_ = 1.26 ± 0.19) did not vary across years (Friedman χ^2^ = 5.298, df = 2, *p* = 0.071). The frequency distribution (Figure S2) of the species-by-species number of “gained” or “lost” plots (i.e., the number of plots where a species was formerly present or absent) was skewed to the right for 1978–2009 community (with a median of 1 gained plot), but was skewed to the left for both the 1978–2019 and 2009–2019 comparisons (with lost plot median of 0.5 and 1 respectively). In detail, comparing 1978 and 2009 communities, 35 species increased the number of occupied plots, 26 decreased and 8 remained stable; comparing 1978 and 2019, 28 species increased, 34 decreased and 5 remained stable; comparing 2009 and 2019, 16 species increased the number of occupied plots, 34 decreased and 11 remained stable (Figure 3).

Considering only species common to all years, the mean number of occupied plots was higher in 2009 (N_1978_ = 2.17 ± 0.31, N_2009_ = 3.61 ± 0.40, N_2019_ = 2.69 ± 0.35; Friedman χ^2^ = 7.6901, df = 2, *p* = 0.02138) and pairwise differences were significant (DunnTest 2009–1978 z = 2.700, *p =* 0.021). In 2009, we observed an increase in the number of species present in more than 50% of the plots, since this value changed from 3 species in 1978 to 13 species in 2009 to decrease again to 6 species in 2019.

**Figure 4:**
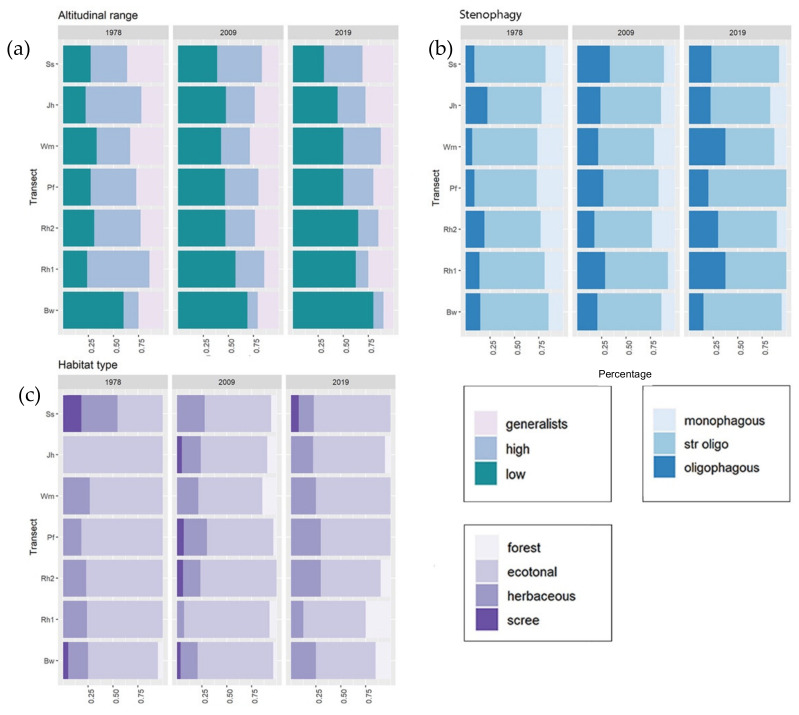
Proportion of species per plot regarding (**a**) preferences in elevation (**b**), stenophagy and (**c**) habitat preferences.

Concerning ecological requirements, for each plot we observed a significant increase in the proportion of low-altitude species in 2009 and 2019 compared to 1978 (Figure 4a; ANOVA F = 6.814, df = 2, *p =* 0.01, mean_1978_ = 32.097 ± 4.866, mean_2009_ = 46.098 ± 4.154, mean_2019_ = 49.114 ± 7.466), and a marginally significant increase in the number of plots occupied by low-altitude species common to all three years (mean_1978_ = 1.42 ± 0.19, mean_2009_ = 2.92 ± 0.45, mean_2019_ = 1.92 ± 0.36; Friedman χ^2^ = 5.706, df = 2, *p =* 0.058). The results showed a decrease in the proportion of generalist species (ANOVA F = 3.092, df = 2, *p =* 0.0826; mean_1978_ = 25.936 ± 7.346, mean_2009_ = 24.632 ± 5.287, mean_2019_ = 17.841 ± 5.686). Although not significant, a decrease in the number of plots occupied by high-altitude species was pinpointed since 1978 (mean_1978_ = 2.35 ± 0.37, mean_2009_ = 2.00 ± 0.55, mean_2019_ = 1.15 ± 0.44, Friedman χ^2^ = 0.087, df = 2, *p =* 0.957). During the last 40 years, a significant decrease in the proportion of monophagous species was observed (Figure 4b; ANOVA F = 19.14, df = 2, *p* < 0.001; mean_1978_ = 21.638 ± 1.705, mean_2009_ = 15.422 ± 2.152, mean_2019_ = 7.446 ± 2.354; Pairwise *t*-test 1978–2009 *p =* 0.05, 1978–2019 *p* < 0.001, 2009–2019 *p =* 0.022), coupled with a drop in the number of plots occupied by monophagous species (mean_1978_ = 1.85 ± 0.44, mean_2009_ = 1.69 ± 0.57, mean_2019_ = 0.61 ± 0.33, Friedman, χ^2^ = 6.0476, df = 2, *p* = 0.049). In parallel, data showed an increase in the proportion of oligophagous species from 1978 (Figure 4b; ANOVA F = 6.399, df = 2, *p* = 0.013; mean_1978_= 13.655 ± 2.167, mean_2009_= 24.592 ± 2.016, mean_2019_= 26.584 ± 3.241; Pairwise *t*-test 1978–2009 *p* = 0.01, 1978–2019 *p* = 0.006) as well in the number of plots they occupied (mean_1978_ = 1.33 ± 0.51, mean_2009_ = 2.92 ± 0.60, mean_2019_ = 2±0.33, Friedman χ^2^ = 7, df = 2, *p* = 0.03).

No significant difference was found when considering habitat preferences (Figure 4c), even though forest species were virtually absent in the 1978 communities (except for the bw plot).

**Figure 5:**
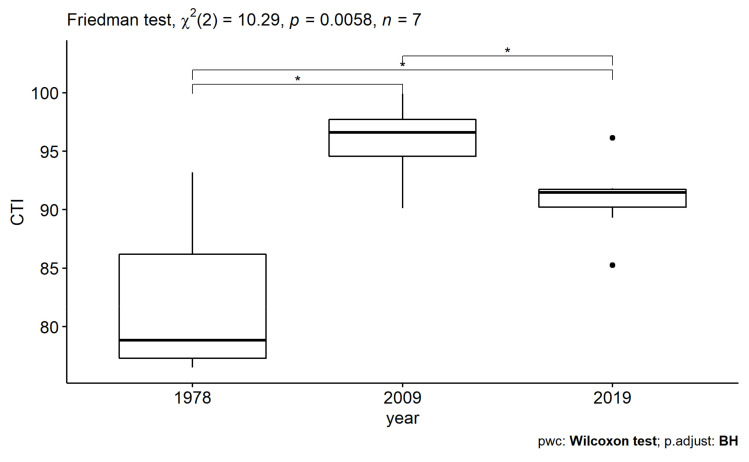
Boxplot of CTI (expressed as °C, multiplied per 10) for the three years of survey. Boxplots show median, quartile, maximum and minimum values; outliers are black dots. Differences in CTI were tested between each sampling year. Asterisks indicate significance in pairwise Wilcoxon test (*p =* 0.047) with Benjamin–Hochberg adjustment.

We observed a general increase in CTI (Figure 5; Friedman χ^2^ = 10.285, df = 2, *p =* 0.005) from 1978. Significant differences were found between CTI of all pairwise years tests (Wilcoxon_1978–2009_ z = 0; *p =* 0.047; Wilcoxon_1978–2019_ z = 2; *p =* 0.047; Wilcoxon_2009–2019_ z = 27; *p =* 0.047), revealing a significant trend towards thermophily in the first period, followed by a slight reversal in the last decade.

**Figure 6:**
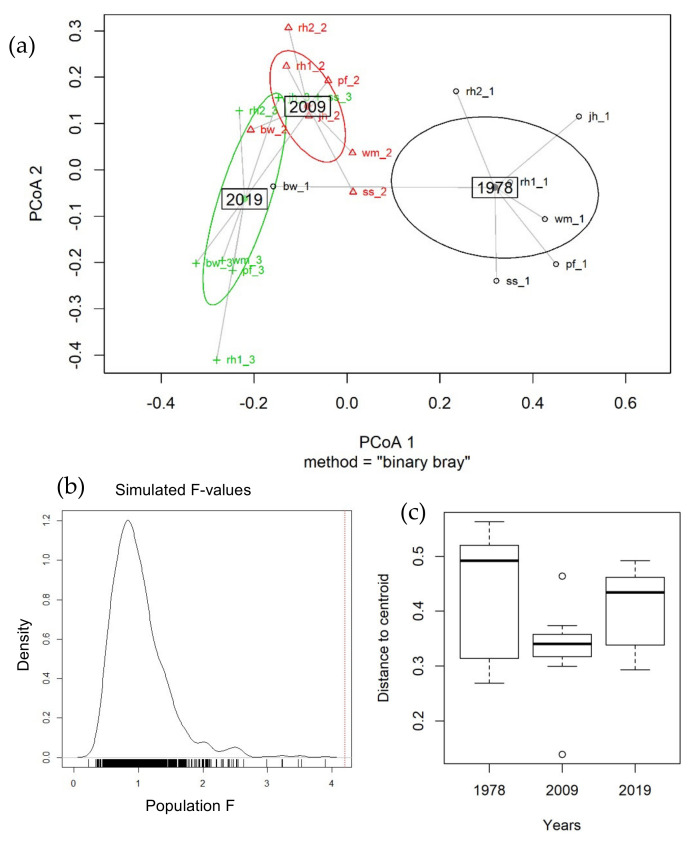
(**a**) Principal Coordinates Analysis of butterfly communities sampled in 1978 (black circles), 2009 (red triangles) and 2019 (green crosses). Each sampling site is connected to its group centroid; ellipses show 95% confidence intervals. (**b**) Simulated and real F-values, after 999 permutations. The distribution of simulated F-values is shown as density plot; the red line shows the real F-value, which is significantly higher 0than all simulated results. (**c**) Analysis of dispersion in community composition. Boxplot of distances of each sampling site from its group centroid for 2019, 2009 and 1978 communities. Boxplots show median, quartile, maximum and minimum values; outliers are open circles.

Species composition differed across years (non-parametric MANOVA F = 4.198, df = 2, R^2^ = 0.298, *p* = 0.001; Figure 6a,b). We observed a reduction in the relative dispersion around centroids only for 2009, due to a decrease in the variability of ecological structure in that year, but differences were not significant (ANOVA F = 1.817, *p =* 0.187). Mean distances of single-year plots from centroids were 0.427 ± 0.047 in 1978, 0.328 ± 0.036 in 2009 and 0.403 ± 0.03 in 2019 (Figure 6c).

Regarding indicator species, IndVal analyses show that 5 species reached the highest value in the 1978 community, 3 during 2009, while only 1 for the community sampled in 2019. Species with IndVal > 0.6 at least in one comparison are shown in Table 3.

#### 3.3.2. Short-Time Analysis (2009–2019)

When we compared the data collected in the whole sampling period between 2009 and 2019, we found that species richness per plot was higher in 2009 (S_2009_ = 35.7 ± 3.1; S_2019_ = 31.6 ± 2), but differences were not significant (*t*-test t = 1.619, df = 6, *p =* 0.156). The number of occupied plots (N_2009_ = 2.94 ± 0.24, N_2019_ = 2.6 ± 0.26) did not vary in the last ten years (Wilcoxon test W = 1434.5, *p =* 0.06). Considering the species occurring both in 2009 and 2019, on average, we observed a higher number of plots occupied in 2009 (N_2009_ = 3.84 ± 0.27, N_2019_ = 3.69 ± 0.29), although the difference was not significant (V = 404.5, *p =* 0.417). Additionally, the number of “gained” or “lost” plots did not change between the two sampling periods (median value was equal to zero), although in 2019 a decrease has been recorded for 20 species (17 species increased the number of occupied plots and 18 remained stable). In 2019, we observed a decrease in the number of species present in more than 50% of the plots, which changed from 34 species in 2009 to 27 species in 2019. Focusing on ecological requirements, the results showed only a marginal decrease in generalist species (*t*-test t = 2.075, df = 6, *p* = 0.083; mean_2009_ = 23.34 ± 0.8, mean_2019_ = 19.39 ± 2). Moreover, no differences in the number of plots occupied in relation to ecological requirements were found, either when considering all or just the species in common between 2009 and 2019. The Shannon index for each surveyed plot revealed no differences between the two years (*t*-test t = 0.081, df = 6, *p* = 0.938). There are differences in species composition between 2019 and 2009 (ANOVA F = 3.317, df = 1, R^2^ = 0.180, *p =* 0.016), but we did not observe significant differences in the dispersion around centroids. Mean distances from centroids of single yearly plots were 0.242 ± 0.02 in 2019, 0.265 ± 0.031 in 2009.

Focusing on indicator species, IndVal analyses show that 2 species (*Melitaea diamina*, *Pontia daplidice*) reached the highest value in the 2009 community and only 1 (*Polyommatus eros*) in 2019, while 40 species were indicators of both years. Species with an IndVal > 0.6 for each category are shown in Table S3. Looking at rarity, in 2009 we detected 28 species (37% of total species) with rr index above the average (rr = 0.63 measured for all 76 species), while 30 species (46% of total, n = 65) were above the rr average (rr = 0.69) in 2019, identifying an increase in the proportion of rare species. The rr indices calculated for the butterfly community in 2009 and 2019 showed a slightly significant difference (Wilcoxon test W = 2927, *p =* 0.05). The psi index, which refers to population size, significantly decreased in 2019 (Wilcoxon test W = 3149.5, *p =* 0.004).

## 4. Discussion

### 4.1. Large Scale Drivers

Ecosystems facing the coldest temperature conditions are most vulnerable to rapid climate change. These include, among others, alpine ecosystems [85]. Here, climate acts as a driver for organism distribution, altering species assemblages. Recent analyses underlined how even a moderate increase in temperature (1 °C) could lead to significant changes in arthropods community composition and species richness [49]. Our analysis of changes observed in the butterfly communities of the SW Italian Alps between 1978, 2009 and 2019 suggests the existence of scale-dependent patterns. We observed a general trend of communities changing according to large scale effects (i.e., climate change). At the same time, small-scale effects (i.e., local vegetational changes and geomorphic processes) can also potentially influence butterfly communities, acting synergistically or constituting a buffer effect.

In the study area, we observed a dramatic increase in temperatures. Using data from Open Data ARPA NWOI dataset, we identified a positive linear trend in the maximum temperatures, which could be quantified in a total increase of 2 °C during the last 40 years (+0.05 °C per year). In contrast, no clear trend was detected in precipitation patterns, and minimum temperatures practically did not vary. Such observed patterns correspond rather well to the trends in climatic variables summarised by Ciccarelli [5] and by Acquaotta [6], although these works observed shallower increases in maximum temperatures (respectively +0.023 °C and +0.018 °C per year) compared to our findings. A reason for this may be that these authors analysed the whole western Alpine region, encompassing wider altitudinal and latitudinal ranges. Instead, we focused on smaller and finer scale, at a level relevant for butterfly communities. Still, since even a small increase in maximum temperatures has potential implications on the composition of butterfly assemblages, by changing the distributional limits of some species, or by promoting the expansion or the local extinction of others [86], it is not surprising that in our study area this temperature increase impacted the biodiversity of the whole butterfly community, mainly in terms of species composition according to their functional traits.

### 4.2. Small Scale Drivers 

A generalised spread of forested areas (open woodland and dense woodland) and a reduction in the size of grasslands during the past decades has been observed for all our study areas. Heathland expansion on the previous grasslands was paralleled by the closure of the canopy layer of the larch forest, accompanied by changes in dominant species. In particular, we observed an increase in some small and tall shrubs species, which were by far less represented in the few qualitative observations from 1978 (e.g., *Juniperus nana*, *Laburnum anagyroides*) and a decrease in abundance of *Rhododendron ferrugineum*. The areas less subjected to the forest cover increase are located near the last remaining centre of pastoral activity (rh2, wm, pf). In all other transect areas, we observed a marked growth of woodland occupancy, with the exception of ss, due to the absence of soil in the block scree.

In the whole study area, and especially in two of the transects (rh1, rh2), we detected cyclic habitat modifications due to periodic avalanches. In the last decade, we observed a high abundance of *Rubus idaeus* in these sites, mainly represented by one-year stems only. The spread and survival of raspberry in the study areas was certainly facilitated by geomorphic disturbance, as known for opportunist species of the subalpine and alpine communities [23,87]. In the European Alps, the effect of climate change, a large-scale driver, is confounded by local-scale human activities. Cattle grazing in the alpine pastures has decreased throughout the last century, allowing a fast recolonisation by trees and shrubs, but this can also result from climatologic variation [21,25,54,88].

### 4.3. Changes in Butterfly Assemblages

The modifications observed in the composition of species assemblages apparently go in the direction of the predicted impacts of temperature increase. In particular, we wish to stress the loss or strong decline in a number of species whose characteristics make them potentially sensitive to climate change, e.g., the geographically localised, the poor dispersers or the ecologically highly specialised butterflies [89,90]. At the same time, we also reported the expansion of some species, such as woodland, thermophilous and generalist, as already recorded at the European scale and generally associated with a global increase in temperature [14,46,54,91,92]. More in detail, since 1978 we observed: (i) a continuous increase in maximum temperatures (Figure 1); (ii) an increase of forest coverage paralleled by a decrease of grasslands (Figure 2); (iii) changes in community compositions (Figure 6) determined by non-random turnover.

Communities sampled in 1978 significantly differed from those sampled in 2009 and in 2019. We observed the loss of specialised species (i.e., *Coenonympha glycerion, Colias phicomone, Pieris callidice*). At the same time a higher number of the truly alpine species (i.e., those only flying above or at the tree line, such as *Colias phicomone* or *Pieris bryoniae*) were only observed in 1978. Conversely, the last decade (2009–2019) shows an increase in relatively thermophilous elements (i.e., *Satyrus ferula*, *Parnassius apollo*) and in shrubland/woodland species (i.e., *Argynnis paphia*, *Brenthis daphne*). Among the species protected at European level by the Habitats Directive (92/43/EEC), *P. apollo* and *P. mnemosyne* showed changes in occupancy and flight period, respectively. Both these two charismatic species are suffering decline in other parts of Italy [93]. Significant results of non-parametric MANOVA (Figure 6a,b) show that butterfly communities have changed across years and that species compositions recorded in 2019 and 2009 were not analogous to those observed in 1978, but represent a reassembly of the original species along with the ingression of several new elements (only 23 species to 77 were found in all years). Indeed, many species showed no stable trend through time, due to changes in occupancy, elevation or phenology (Figure 3 and Figure 4). Nevertheless, despite the continuous increase of temperatures, the comparison between communities sampled in 2009 and 2019 shows minor differences (Figure 6c) in the position of their centroids. This seeming local resilience is mainly due to the inflow of 11 ‘new’ and relatively generalist species in the short period (2009–2019). More in general, we noticed a tendency towards biotic homogenisation referred to the increase in biological similarity among communities (*sensu* [94]). Group variances between years were not significantly different (see the multivariate analogous of the Levene’s test in 3.3.1 result section). However, a reduction in the distinctiveness of sampled communities has been observed, in particular from 1978 to 2009 (Figure 6).

Even if studies on the community-level responses of butterflies through time are less frequent than analyses of single species responses, results similar to ours are paving common routes and patterns in butterfly ecology. For example, a similar change in community composition over time, along with an increase in community similarity, has been observed in the data analysis from the UK Butterfly Monitoring Scheme over a 20-year period [95]. In this latter case, the main causes were related to an increase in southerly distributed species, particularly the more generalist ones, which spread northwards, accompanied by the decline of some localised species. In mountain ecosystems, Wilson et al. [92] observed that widespread species became increasingly dominant during the last 40 years, while high-altitude species strongly declined. Similar patterns were observed along the altitudinal gradient in the space-for-time studies even on a short time gap [60]. Climate change acts by filtering vulnerable species as observed for habitat simplification [96], but this phenomenon was already observed as a consequence of habitat deterioration, such as in the case of agricultural intensification [97]. This rearrangement at the community level is accompanied by some small changes in the relative representation of some ecological groups. Looking at the ecological categories of the sampled species, we can observe that differences are mainly related to an increase in the proportion of low-altitude species and changes related to habitat specialisation. Species linked to the wooded habitats strongly increased in frequency, apparently at the expenses of species linked to grasslands occurring above the tree line (subalpine and alpine belts). We can also observe a generalised ingression of widespread and common species (e.g., *Ochlodes sylvanus*, *Aglais urticae*, *Papilio machaon*, *Colias crocea*, *Pieris brassicae*; see Figure 3 and Table S1), mainly characterised by high vagility and broad ecological tolerance, as well as a general spread of thermophilous species, as confirmed by an increase in the Community Temperature Index (Figure 5).

More interestingly, the analysis of indicator species by the IndVal method (Table 3) and the comparison of the list of species observed in 1978 with the full dataset of 2009 (Figure 3, Table S1) identifies some ‘winner’ species (the expanding ubiquitous species in our dataset), as opposed to others that may be viewed as ‘losers’ (replaced species) [98]. In particular two species were completely absent from the 1978 dataset and were present in most of the plots sampled in 2009: *Argynnis paphia* (in 5 of 7 plots) and *Brenthis daphne* (in all plots). These species are characterised by a long flight period, spanning from the beginning of July until the middle of August, and would certainly have been recorded, if at all present, in 1978. Both species have undergone, during the past decades, a northwards expansion accompanied by a stable southern boundary [91,99,100], while an increase in the elevational optimum has also been recorded in the case of *Argynnis paphia* [8]. *Argynnis paphia* is a woodland butterfly, which uses as larval host plant some *Viola* species, but lays its eggs in the tree bark, where the newly hatched larvae hibernate, and selects sites where well-shaded larval host plants are abundant. *Brenthis daphne* is a sub-nemoral, scrubland species and uses as larval host plants many species of *Rubus*. Brambles are early successional, highly competitive, fast-growing species that are expanding everywhere in Europe, being generally favoured by an abundant nitrogen supply; in mountain ecosystems, they can quickly occupy places set free from avalanches [101,102]. 

To come now to the ‘losers’, we observed three cases of apparent species substitution (indicating with this term the complete disappearance of one species, replaced by a congeneric one since 1978) at our sampling sites, indicating a general loss of specialised and narrow-range species and a general expansion of ecologically tolerant elements. In the last decade, we neither recorded *Coenonympha darwiniana*, an alpine endemic, nor *Coenonympha glycerion*, a hygrophilous species, but we found *Coenonympha arcania*, that had not been recorded in 1978. The latter is a widespread species, characteristic of the lowland ecotonal habitats and a much stronger generalist than the previous two. A similar case is that of *Colias phicomone*, a xerophilous species, linked to open herbaceous habitats occurring above the tree line (subalpine and alpine belts). It was repeatedly found in 1978 but was substituted in 2009 by the ingression of *Colias crocea*, an ecotonal, generalist and rather thermophilous species, characterised by very high vagility. Finally, *Pieris callidice*, a microthermic, xerophilous species of the open herbaceous environments of the alpine belt, has disappeared completely from the study sites, where we found *Pieris daplidice*, a generalist, thermophilous species, typical of the low elevations. *Colias phicomone* and *Pieris callidice* did not show any altitudinal shift since they have not been found in higher elevations plots sampled in 2019.

As a word of caveat, we note that one source of bias in our analysis is represented by the reduced sampling period in 1978. The strong observed differences in community composition might be reduced if sampling data from 1978 were available for all the favourable season (June–August), allowing to disentangle real differences in community composition from simpler changes in the flight period of individual species. To overcome this, we selected a much longer time frame for samplings carried out in 2009 and 2019, so that species having anticipated their flight period would have been detected anyway, but of course we could not use the same procedure for 1978. In the latter years, some species might have flown before, or perhaps later, than our sampling period. This is, however, unlikely. On the one hand, our climatic analysis showed that 1978 was colder than 2009, and species would not tend to fly earlier in such conditions. On the other hand, early to mid-August is the end of the flying period of butterflies throughout the Italian Alps, with very few exceptions. In any case, such a reduced sampling period (end of July-beginning of August) represents the peak of activity for butterfly communities in the mountain ecosystems of the Alps. Moreover, the prolonged sampling period in 2009 and 2019, which covered the whole favourable season, has allowed to reveal that some (at least 8) species have completely disappeared. Secondly, we observed that most species present in the reduced dataset of 2009 and 2019 (end of July-beginning of August, used for the statistical comparisons) and missing from the 1978 dataset are species characterised by long flight periods, centred in the middle of summer. Consequently, they should have been found, if present, also in 1978. Such observations confirmed the robustness of our results and the reliability of changes in community compositions through time.

## 5. Conclusions

Our work represents the first attempt to describe changes in butterfly community composition in the European Alps over a long-term temporal scale. Responses to climate and habitat changes vary widely among species occurring in the same communities, and consequently it is important to understand the heterogeneity of species responses and its implication on changes in community compositions. However, it is difficult to assess the relative role of climate and vegetation modifications in determining the observed pattern in butterfly communities. The observed sharp increase in vagile, tolerant and thermophilous species perfectly fits the consequence of an increase in temperature ([41,57,92,103,104] and references therein). The reduction of open areas, in fact, is not a problem for the highly mobile species that can easily reach another suitable site, while the less mobile species will remain *locked* to their continuously shrinking micro-environment [105,106]. In the same way, because of their strong adaptability, the broadly tolerant species will take advantage of a *changing environment*, to the detriment of the stenotopic and more specialised species, thereby contributing to reduce the community distinctiveness [106,107].

Species respond individually to the changing environmental conditions, depending mainly on their functional traits and habitat requirements [108,109]. This can originate new species assemblages, which can be appreciated only by the examination of entire communities throughout time (e.g., [92,110,111]). We observed the highest changes during the first time frame (1978–2009), while in the last decade a slow and background erosion of species emerged. In this framework, we can envisage that long-term environmental change can enhance the observed pattern of alterations in butterfly guild compositions consistently with predicted impact by local warming.

Several studies investigating community changes over time relied on comparisons between current data and historical data sets (atlases, collection specimens), the latter of which were generally collected in a non-standardised way and/or referred to a much coarser spatial grain [112]. Transects set in well-specified areas represent a more appropriate tool, e.g., [60]. The implementation of projects aiming to record and share data on butterfly observations, such as the Italian Butterfly Monitoring Scheme (https://butterfly-monitoring.net/it (accessed on 23 November 2021), will guarantee the availability of robust and standardised data for research addressing long- or short-term butterfly assemblage variations.

## Figures and Tables

**Table 1:** Habitat type, elevation and coordinates (WGS84) of the seven plots monitored in 1978, 2009 and 2019.

Habitat Type	Code	Elevation (m)	Coordinates
Beech-wood clearings	Bw	1250	44°12′56.62″−7°16′48.2″
*Rhododendron* heathland	rh1	1600	44°12′11.62″−7°14′53.81″
*Rhododendron* heathland	rh2	1750	44°12′7.75″−7°14′30.27″
Wet acid grassland	Wm	1750	44°12′2.77″−7°14′24.07″
Subalpine screes	Ss	1900	44°11′21.91″−7°13′18.34″
*Juniperus* heathland	Jh	1800	44°11′51.2″−7°13′38.32″
Pastures with *Festuca paniculata*	Pf	1750	44°12′6.72″−7°14′23.99″

Code represents the abbreviation of each habitat type, as used in the main text.

**Table 2:** Climatic variables calculated for 1978, 2009 and 2019.

	1978	2009	2019
T mean	4.07	5.19	5.67
T mean (DJF)	−2.34	−3.06	−0.07
T mean (MAM)	1.71	4.21	2.49
T mean (JJA)	10.77	13.25	14.12
MTCO (Jan)	−3.65	−3.58	−2.85
dd (Feb)	0	0	88.13
dd (Apr)	7.14	5.7	241.62
dd (Jun)	277.35	676.6	243.98
dd (Aug)	1100.48	1824.47	3734.76

All data are expressed as Celsius degrees (°C). T mean = mean annual temperature; T mean (DJF) = mean winter temperature (December-January-February); T mean (MAM) = mean spring temperature (March-April-May); T mean (JJA) = mean summer temperature (June-July-August); MTCO (Jan) = Minimum Temperature of the Coldest Month, January; dd = accumulated growing degree days with a base temperature of 5 °Cfrom January until February (Feb), April (Apr), June (Jun), and August (Aug).

**Table 3:** Indicator species (IndVal species) for the three sampling periods.

Species	1978	2009	2019	1978–2009	1978–2019	2009–2019	All	*p*-Value	
*Anthocharis cardamines*	**0.845**	0.000	0.000	0.598	0.598	0.000	0.488	0.004	1978
*Parnassius mnemosyne*	**0.756**	0.000	0.000	0.535	0.535	0.000	0.436	0.015	
*Pieris bryoniae*	0.655	0.000	0.000	0.463	0.463	0.000	0.378	0.074	
*Pontia callidice*	0.655	0.000	0.000	0.463	0.463	0.000	0.378	0.083	
*Pyrgus carthami*	**0.845**	0.000	0.000	0.598	0.598	0.000	0.488	0.004	
*Aricia nicias*	0.134	**0.668**	0.267	0.567	0.283	0.661	0.617	0.135	2009
*Parnassius apollo*	0.154	**0.617**	0.154	0.546	0.218	0.546	0.535	0.264	
*Polyommatus icarus*	0.154	**0.772**	0.000	0.655	0.109	0.546	0.535	0.017	
*Polyommatus bellargus*	0.000	0.000	**0.655**	0.000	0.463	0.463	0.378	0.075	2019
*Eumedonia eumedon*	0.668	0.401	0.000	**0.756**	0.472	0.283	0.617	0.054	
*Lycaena eurydame*	0.617	0.309	0.000	**0.655**	0.436	0.218	0.535	0.147	1978–2009
*Melitaea athalia*	0.286	0.571	0.143	**0.606**	0.303	0.505	0.577	0.605	
*Plebejus argus*	0.571	0.429	0.000	**0.707**	0.404	0.303	0.577	0.125	
*Polyommatus escheri*	0.286	0.571	0.143	**0.606**	0.303	0.505	0.577	0.612	
*Vanessa cardui*	0.378	0.756	0.000	**0.802**	0.267	0.535	0.655	0.027	
*Argynnis niobe*	0.000	0.429	0.571	0.303	0.404	**0.707**	0.577	0.124	2009–2019
*Argynnis paphia*	0.000	0.630	0.504	0.445	0.356	**0.802**	0.655	0.021	
*Brenthis daphne*	0.000	0.630	0.504	0.445	0.356	**0.802**	0.655	0.019	
*Coenonympha arcania*	0.000	0.571	0.429	0.404	0.303	**0.707**	0.577	0.105	
*Erebia ligea*	0.143	0.429	0.429	0.404	0.404	**0.606**	0.577	0.607	
*Lycaena alciphron*	0.143	0.429	0.429	0.404	0.404	**0.606**	0.577	0.600	
*Lycaena virgaureae*	0.275	0.642	0.642	0.648	0.648	**0.907**	0.900	0.009	
*Satyrus ferula*	0.114	0.570	0.570	0.483	0.483	**0.806**	0.724	0.036	
*Speyeria aglaja*	0.239	0.478	0.478	0.507	0.507	0.676	**0.690**	NA	All
*Boloria titania*	0.478	0.478	0.239	0.676	0.507	0.507	**0.690**	NA	
*Erebia albergana*	0.567	0.661	0.283	0.869	0.601	0.668	**0.873**	NA	
*Erebia euryale*	0.314	0.629	0.419	0.667	0.519	0.741	**0.787**	NA	
*Erebia melampus*	0.267	0.267	0.535	0.378	0.567	0.567	**0.617**	NA	
*Lasiommata maera*	0.359	0.598	0.239	0.676	0.423	0.592	**0.690**	NA	
*Pieris rapae*	0.550	0.458	0.550	0.713	0.778	0.713	**0.900**	NA	
*Thymelicus lineola*	0.239	0.598	0.359	0.592	0.423	0.676	**0.690**	NA	

Values were tested by 999 permutations. Species having a *p*-value below 0.05 are underlined. The highest value obtained for each species is shown in bold.

## Data Availability

Data are available on request from the corresponding author.

## Supplementary Materials

The following are available online at https://www.mdpi.com/article/10.3390/insects13010043/s1, Table S1: Butterfly community sampled in 1978, 2009 and 2019; Table S2: Information about sampling events; Table S3: Indicator species (IndVal species) for both communities; Figure S1: Location and altitudinal gradient of sites surveyed by transects (pollard walk) in the Valasco Valley (SW Italian Alps); Figure S2: Frequency distribution of the species-by-species number of “gained” or “lost” plots.
