# Supplementary material for: Changes in Alpine Butterfly Communities during the Last 40 Years
Source: Insects. 2021 Dec 30;13(1):43. doi: 10.3390/insects13010043 (PMC8778691; doi:10.3390/insects13010043)

Table S1. Butterfly community sampled in 1978, 2009 and 2019.

Family	Species*	IUCN assesment			Stenophagy	Habitat requirements	Altitudinal preferences	1978**	2009***	2019***
		Europe; EU27	Mediterrenean	Italy						
Hesperiidae	*Carcharodus flocciferus*	NT; LC		LC	SO	E	L	x	1	1
	Erynnis tages			LC	M	E	G			1
	Hesperia comma			LC	SO	H	L		1	1
	Ochlodes sylvanus			LC	SO	E	L		11	6
	Pyrgus carlinae	LC	LC	LC	O	H	L		5	8
	Pyrgus carthami			LC	M	E	L	x	6	
	Pyrgus malvoides	LC	LC	LC	M	E	G	x		3
	Spialia sertorius			LC	SO	E	L		1	
	Thymelicus lineola			LC	SO	E	L	x	19	10
	Thymelicus sylvestris			LC	SO	E	L	x	4	11
Lycaenidae	*Aricia agestis*			LC	M	E	L		1	1
	Aricia allous			LC	O	E	H		4	
	Aricia nicias	LC		LC	O	E	H	x	17	26
	Callophrys rubi			LC	M	E	G	x		
	Celastrina argiolus			LC	M	F	L			1
	Cupido minimus			LC	SO	H	G			1
	Cupido osiris			LC	SO	E	L			4
	Cyaniris semiargus			LC	SO	E	L	x	9	6
	Eumedonia eumedon			LC	O	E	H	x	17	17
	Lycaeides idas			LC	SO	H	L	x	4	1
	Lycaena alciphron	LC; NT		LC	O	E	L	x	5	10
	Lycaena eurydame			LC	SO	E	H	x	14	1
	Lycaena phlaeas			LC	O	E	G		1	
	Lycaena subalpina			LC	O	E	H		8	
	Lycaena virgaureae			LC	SO	E	H	x	234	94
	Maculinea arion	EN		LC	SO	E	L		4	6
	Plebejus argus			LC	SO	H	G	x	72	11
	Polyommatus bellargus			LC	SO	H	L			6
	Polyommatus coridon			LC	SO	H	L	x	69	5
	Polyommatus dorylas	NT		LC	SO	H	L			3
	Polyommatus eros	NT		LC	SO	H	L			9
	Polyommatus escheri			LC	O	E	L	x	43	9
	Polyommatus icarus			LC	SO	E	L	x	70	10
	Polyommatus thersites			LC	O	H	L			1
Nymphalidae	*Aglais urticae*			LC	O	H	G		11	5
	Argynnis niobe	LC; NT		LC	O	H	L		9	26
	Argynnis paphia			LC	O	F	L		49	26
	Boloria euphrosyne			LC	M	E	L	x	40	39
	Boloria graeca	LC		NT	O	H	H		1	1
	Boloria titania	NT; LC		LC	M	E	H	x	60	100
	Brenthis daphne			LC	SO	E	L		40	19
	Coenonympha arcania			LC	SO	E	L		32	36
	Coenonympha darwiniana			LC	SO	H	H	x		
	Coenonympha glycerion			LC	SO	H	L	x		
	Coenonympha pamphilus			LC	SO	H	L			1

	Erebia aethiops			LC	SO	F	L			4
	Erebia albergana			LC	SO	E	H	x	88	28
	Erebia epiphron			LC	SO	E	H	x	1	1
	Erebia euryale			LC	SO	E	H	x	190	59
	Erebia ligea			LC	SO	F	L	x	8	36
	Erebia manto			LC	SO	E	H	x		
	Erebia melampus			LC	SO	E	H	x	10	19
	Erebia meolans			LC	SO	S	H	x	5	11
	Erebia montana			LC	SO	S	H	x	1	8
	Erebia neoridas			LC	SO	E	H		12	2
	Erebia triaria			LC	SO	H	H	x	1	1
	Hyponephele lycaon			LC	SO	E	L		26	3
	Inachis io			LC	SO	E	L	x		
	Issoria lathonia			LC	M	H	L		1	
	Lasiommata maera			LC	SO	E	L	x	53	54
	Lasiommata megera			LC	SO	S	G		1	
	Lasiommata petropolitana			LC	SO	E	H		2	
	Maniola jurtina	LC		LC	SO	E	L	x	16	1
	Melanargia galathea			LC	O	E	L	x	5	25
	Melitaea athalia			LC	M	E	L	x	63	49
	Melitaea cinxia			LC	M	E	L	x	5	6
	Melitaea deione			LC	O	E	G			1
	Melitaea diamina	LC; NT		LC	M	E	L		5	
	Oeneis glacialis			LC	O	S	H		2	
	Pararge aegeria			LC	SO	W	G		2	
	Polygonia c-album			LC	M	E	L		3	3
	Satyrus ferula			LC	SO	E	L	x	167	11
	Speyeria aglaja			LC	M	E	G	x	27	28
	Vanessa atalanta			LC	SO	E	L	x	15	3
	Vanessa cardui			LC	SO	E	L	x	136	40
Papilionidae	*Iphiclides podalirius*			LC	M	E	L		2	
	Papilio machaon			LC	SO	E	G		5	
	Parnassius apollo	NT		LC	SO	S	L	x	57	24
	Parnassius mnemosyne	NT; LC		LC	O	E	H	x	5	16
Pieridae	*Anthocharis cardamines*			LC	SO	E	G	x	20	27
	Aporia crataegi			LC	SO	E	G	x	11	9
	Colias crocea			LC	SO	E	L		21	
	Colias phicomone	NT		LC	SO	H	H	x		
	Gonepteryx cleopatra			LC	O	E	L		5	7
	Gonepteryx rhamni			LC	SO	E	L	x	9	9
	Leptidea sinapis			LC	O	E	G		1	1
	Pieris brassicae			LC	SO	H	G		20	3
	Pieris bryoniae			LC	SO	E	H	x	1	3
	Pieris mannii			LC	SO	E	L		2	2
	Pieris napi			LC	SO	E	G	x	44	49
	Pieris rapae			LC	SO	H	G	x	89	45
	Pontia callidice			LC	M	H	H	x		
	Pontia daplidice			LC	M	E	L		3	2
Riodinidae	*Hamearis lucina*			LC	SO	E	L	x	2	3

There are no present Italian endemisms in the studied community

* Species names are according to Balletto, E., Cassulo, L. A., & Bonelli, S. (2014). An annotated checklist of the Italian butterflies and skippers (*Papilionoidea, Hesperiioidea*).
(x) Species presence; * Species abundance.

Stenophagy: M-monophagous, SO-strictly olygophagous (one foodplant genus), O-oligophagous (one foodplant family)

Habitat requirements: W-woodland, E-ecotone, H-open herbaceous, S-scree.

Altitudinal preferences: H-high altitude, G-generalist, L-low altitude.

Table S2. Information about sampling events.

Transects	Year	Date	Hours	Weather	Wind	Operator
**bw**	1978	26 July - 8 August	10:00-15:00	Sunny	absent	Emilio Balletto
**rh1**	1978	26 July - 8 August	10:00-15:00	Sunny	absent	Emilio Balletto
**pf**	1978	26 July - 8 August	10:00-15:00	Sunny	absent	Emilio Balletto
**rh2**	1978	26 July - 8 August	10:00-15:00	Sunny	absent	Emilio Balletto
**wm**	1978	26 July - 8 August	10:00-15:00	Sunny	absent	Emilio Balletto
**jh**	1978	26 July - 8 August	10:00-15:00	Sunny	absent	Emilio Balletto
**ss**	1978	26 July - 8 August	10:00-15:00	Sunny	moderate	Emilio Balletto
**rh1**	2009	04/06/09	11:30	Sunny	absent	Lorenzo Fracastoro
**bw**	2009	19/06/09	11:00	cloudy	absent	Lorenzo Fracastoro
**rh1**	2009	19/06/09	12:30	Sunny	absent	Lorenzo Fracastoro
**pf**	2009	19/06/09	13:10	Sunny	absent	Lorenzo Fracastoro
**rh2**	2009	19/06/09	14:20	Sunny	moderate	Lorenzo Fracastoro
**wm**	2009	19/06/09	13:00	Sunny	weak	Lorenzo Fracastoro
**jh**	2009	19/06/09	15:30	cloudy	weak	Lorenzo Fracastoro
**rh1**	2009	29/06/09	12:00	Sunny	absent	Lorenzo Fracastoro
**pf**	2009	29/06/09	13:00	Sunny	absent	Lorenzo Fracastoro
**wm**	2009	29/06/09	13:40	cloudy	weak	Lorenzo Fracastoro
**jh**	2009	29/06/09	11:35	Sunny	weak	Lorenzo Fracastoro
**ss**	2009	29/06/09	12:50	Sunny	weak	Lorenzo Fracastoro
**bw**	2009	07/07/09	11:40	partially cloudy	weak	Lorenzo Fracastoro
**rh1**	2009	08/07/09	15:00	Sunny	absent	Lorenzo Fracastoro
**pf**	2009	08/07/09	14:00	Sunny	absent	Lorenzo Fracastoro
**rh2**	2009	08/07/09	13:00	Sunny	absent	Lorenzo Fracastoro
**wm**	2009	08/07/09	13:30	Sunny	weak	Lorenzo Fracastoro
**jh**	2009	08/07/09	12:30	Sunny	weak	Lorenzo Fracastoro
**ss**	2009	08/07/09	12:30	Sunny	weak	Lorenzo Fracastoro
**bw**	2009	16/07/09	11:00	Sunny	absent	Lorenzo Fracastoro
**rh1**	2009	16/07/09	12.30	Sunny	absent	Lorenzo Fracastoro
**rh2**	2009	16/07/09	16.30	Sunny	moderate	Lorenzo Fracastoro
**wm**	2009	16/07/09	16.30	Sunny	moderate	Lorenzo Fracastoro
**pf**	2009	17/07/09	14.30	Sunny	moderate	Lorenzo Fracastoro
**jh**	2009	17/07/09	13:00	Sunny	absent	Lorenzo Fracastoro
**ss**	2009	17/07/09	12.00	cloudy	absent	Lorenzo Fracastoro
**bw**	2009	24/07/09	13.30	Sunny	absent	Lorenzo Fracastoro
**rh1**	2009	25/07/09	16.45	partially cloudy	absent/moderate	Lorenzo Fracastoro
**pf**	2009	25/07/09	15.40	Sunny/partially cloudy	moderate/strong	Lorenzo Fracastoro
**rh2**	2009	25/07/09	13.40	Sunny	moderate	Lorenzo Fracastoro
**wm**	2009	25/07/09	13.30	Sunny	moderate	Lorenzo Fracastoro
**jh**	2009	25/07/09	11.30	Sunny	absent	Lorenzo Fracastoro
**ss**	2009	25/07/09	10:00	Sunny	absent	Lorenzo Fracastoro
**bw**	2009	29/07/09	12.10	Sunny	moderate	Lorenzo Fracastoro
**rh1**	2009	29/07/09	17.00	cloudy/changeable	absent	Lorenzo Fracastoro
**pf**	2009	30/07/09	16.30	Sunny	moderate	Lorenzo Fracastoro
**rh2**	2009	30/07/09	10.30	Sunny	moderate/absent	Lorenzo Fracastoro
**wm**	2009	30/07/09	16.00	cloudy	moderate	Lorenzo Fracastoro
**jh**	2009	30/07/09	13.30	Sunny	absent/moderate	Lorenzo Fracastoro

**ss**	2009	30/07/09	15:00	Sunny	moderate	Lorenzo Fracastoro
**bw**	2009	06/08/09	14.00	Sunny	moderate	Lorenzo Fracastoro
**rh1**	2009	06/08/09	16.50	Sunny/changeable	absent	Lorenzo Fracastoro
**rh2**	2009	07/08/09	10.15	Sunny	absent/moderate	Lorenzo Fracastoro
**ss**	2009	07/08/09	14.30	cloudy	moderate	Lorenzo Fracastoro
**pf**	2009	08/08/09	13.00	cloudy	moderate	Lorenzo Fracastoro
**wm**	2009	08/08/09	12.30	cloudy	moderate	Lorenzo Fracastoro
**jh**	2009	08/08/09	11.00	changeable	absent/moderate	Lorenzo Fracastoro
**rh2**	2009	16/08/09	11.30	Sunny	absent/moderate	Lorenzo Fracastoro
**bw**	2009	17/08/09	16.00	changeable	absent/moderate	Lorenzo Fracastoro
**rh1**	2009	17/08/09	15.00	cloudy/changeable	absent/moderate	Lorenzo Fracastoro
**pf**	2009	17/08/09	13.30	Sunny	moderate	Lorenzo Fracastoro
**wm**	2009	17/08/09	13.30	changeable	moderate	Lorenzo Fracastoro
**jh**	2009	17/08/09	11.30	Sunny	moderate/absent	Lorenzo Fracastoro
**ss**	2009	17/08/09	10.30	Sunny	moderate/absent	Lorenzo Fracastoro
**bw**	2019	07/06/19	11:30	changeable	weak	Michele Zaccagno
**rh1**	2019	08/06/19	10:55	Sunny	weak	Michele Zaccagno
**pf**	2019	08/06/19	11:45	Sunny	moderate	Michele Zaccagno
**rh2**	2019	08/06/19	12:30	Sunny	absent	Michele Zaccagno
**wm**	2019	08/06/19	13:50	Sunny	weak	Michele Zaccagno
**jh**	2019	08/06/19	14:45	Sunny	absent	Michele Zaccagno
**ss**	2019	08/06/19	15:45	Sunny	weak	Michele Zaccagno
**bw**	2019	19/06/19	11:00	Sunny	absent	Michele Zaccagno
**pf**	2019	19/06/19	14:05	Sunny	medio	Michele Zaccagno
**rh2**	2019	19/06/19	15:00	Sunny	weak	Michele Zaccagno
**wm**	2019	19/06/19	15:35	Sunny	moderate	Michele Zaccagno
**rh1**	2019	20/06/19	16:05	Sunny	moderate	Michele Zaccagno
**jh**	2019	20/06/19	11:35	Sunny	moderate	Michele Zaccagno
**ss**	2019	20/06/19	12:45	light clouds	moderate	Michele Zaccagno
**bw**	2019	27/06/19	10:30	Sunny	absent	Michele Zaccagno
**rh1**	2019	27/06/19	12:40	Sunny	weak	Michele Zaccagno
**pf**	2019	27/06/19	13:30	Sunny	medio	Michele Zaccagno
**rh2**	2019	27/06/19	15:20	Sunny	weak	Michele Zaccagno
**wm**	2019	27/06/19	14:45	Sunny	moderate	Michele Zaccagno
**jh**	2019	27/06/19	16:15	Sunny	absent	Michele Zaccagno
**ss**	2019	28/06/19	15:10	Sunny	moderate	Michele Zaccagno
**jh**	2019	06/07/19	15:40	Sunny	absent	Michele Zaccagno
**ss**	2019	06/07/19	14:50	changeable	absent	Michele Zaccagno
**bw**	2019	07/07/19	10:45	Sunny	absent	Michele Zaccagno
**rh1**	2019	07/07/19	12:30	Sunny	weak	Michele Zaccagno
**pf**	2019	07/07/19	13:15	Sunny	moderate	Michele Zaccagno
**rh2**	2019	07/07/19	15:15	Sunny	moderate	Michele Zaccagno
**wm**	2019	07/07/19	14:45	Sunny	moderate	Michele Zaccagno
**ss**	2019	07/07/19	13:45	changeable	weak	Michele Zaccagno
**bw**	2019	18/07/19	16:00	changeable	absent	Michele Zaccagno
**rh1**	2019	18/07/19	14:50	changeable	absent	Michele Zaccagno
**pf**	2019	18/07/19	14:20	changeable	moderate	Michele Zaccagno
**rh2**	2019	18/07/19	13:45	Sunny	weak	Michele Zaccagno
**wm**	2019	18/07/19	11:10	Sunny	weak	Michele Zaccagno
**jh**	2019	18/07/19	12:10	Sunny	weak	Michele Zaccagno
**ss**	2019	19/07/19	13:45	changeable	weak	Michele Zaccagno
**ss**	2019	31/07/19	11:50	Sunny	moderate	Michele Zaccagno
**bw**	2019	01/08/19	10:20	Sunny	absent	Michele Zaccagno
**rh1**	2019	01/08/19	12:15	Sunny	absent	Michele Zaccagno
**pf**	2019	01/08/19	13:00	Sunny	moderate	Michele Zaccagno
**rh2**	2019	01/08/19	15:00	changeable	absent	Michele Zaccagno

**wm**	2019	01/08/19	14:00	changeable	weak	Michele Zaccagno
**bw**	2019	01/08/19	16:00	light clouds	weak	Michele Zaccagno
**bw**	2019	10/08/19	14:45	Sunny	weak	Michele Zaccagno
**rh1**	2019	11/08/19	11:30	Sunny	absent	Michele Zaccagno
**pf**	2019	11/08/19	12:20	Sunny	moderate	Michele Zaccagno
**rh2**	2019	11/08/19	13:05	changeable	moderate	Michele Zaccagno
**wm**	2019	11/08/19	13:35	changeable	moderate	Michele Zaccagno
**jh**	2019	11/08/19	15:00	changeable	absent	Michele Zaccagno
**ss**	2019	11/08/19	15:45	light clouds	absent	Michele Zaccagno
**bw**	2019	21/08/19	11:10	changeable	moderate	Michele Zaccagno
**pf**	2019	21/08/19	13:00	changeable	moderate	Michele Zaccagno
**rh2**	2019	21/08/19	14:20	changeable	weak	Michele Zaccagno
**jh**	2019	21/08/19	13:50	changeable	absent	Michele Zaccagno
**ss**	2019	21/08/19	13:30	changeable	absent	Michele Zaccagno

Table S3. Indicator species (IndVal species) for both communities. Values were tested by 999 permutations.

Specie	2009	2019	2009-2019	p.value	
Melitaea diamina	**0.655**	0.000	0.463	0.185	***2009***
Pontia daplidice	**0.655**	0.000	0.463	0.191	
Polyommatus eros	0.000	**0.655**	0.463	0.178	***2019***
Aglais urticae	0.504	0.630	**0.802**	NA	***Both***
Anthocharis cardamines	0.655	0.655	**0.926**	NA	
Aporia crataegi	0.401	0.668	**0.756**	NA	
Argynnis niobe	0.535	0.535	**0.756**	NA	
Argynnis paphia	0.504	0.630	**0.802**	NA	
Aricia nicias	0.401	0.668	**0.756**	NA	
Boloria euphrosyne	0.684	0.570	**0.886**	NA	
Boloria titania	0.655	0.655	**0.926**	NA	
Brenthis daphne	0.734	0.629	**0.964**	NA	
Carcharodus floccifer	0.463	0.463	**0.655**	NA	
Coenonympha arcania	0.598	0.598	**0.845**	NA	
Colias crocea	0.463	0.463	**0.655**	NA	
Cyaniris semiargus	0.504	0.630	**0.802**	NA	
Erebia albergana	0.707	0.707	**1.000**	NA	
Erebia euryale	0.684	0.570	**0.886**	NA	
Erebia ligea	0.570	0.684	**0.886**	NA	
Erebia melampus	0.535	0.535	**0.756**	NA	
Eumedonia eumedon	0.570	0.684	**0.886**	NA	
Gonepteryx rhamni	0.655	0.655	**0.926**	NA	
Lasiommata maera	0.655	0.655	**0.926**	NA	
Lycaena alciphron	0.504	0.630	**0.802**	NA	
Lycaena virgaureae	0.707	0.707	**1.000**	NA	
Maculinea arion	0.571	0.429	**0.707**	NA	
Melitaea athalia	0.707	0.707	**1.000**	NA	
Melitaea cinxia	0.463	0.463	**0.655**	NA	
Papilio machaon	0.309	0.617	**0.655**	NA	
Parnassius apollo	0.546	0.764	**0.926**	NA	
Parnassius mnemosyne	0.535	0.535	**0.756**	NA	
Pieris brassicae	0.630	0.504	**0.802**	NA	
Pieris napi	0.629	0.734	**0.964**	NA	
Pieris rapae	0.707	0.707	**1.000**	NA	
Plebejus argus	0.401	0.668	**0.756**	NA	
Polyommatus coridon	0.630	0.504	**0.802**	NA	
Polyommatus escheri	0.429	0.571	**0.707**	NA	
Polyommatus icarus	0.717	0.478	**0.845**	NA	
Satyrus ferula	0.570	0.684	**0.886**	NA	
Speyeria aglaja	0.629	0.734	**0.964**	NA	
Thymelicus lineola	0.401	0.668	**0.756**	NA	
Thymelicus sylvestris	0.571	0.429	**0.707**	NA	
Vanessa cardui	0.734	0.629	**0.964**	NA	

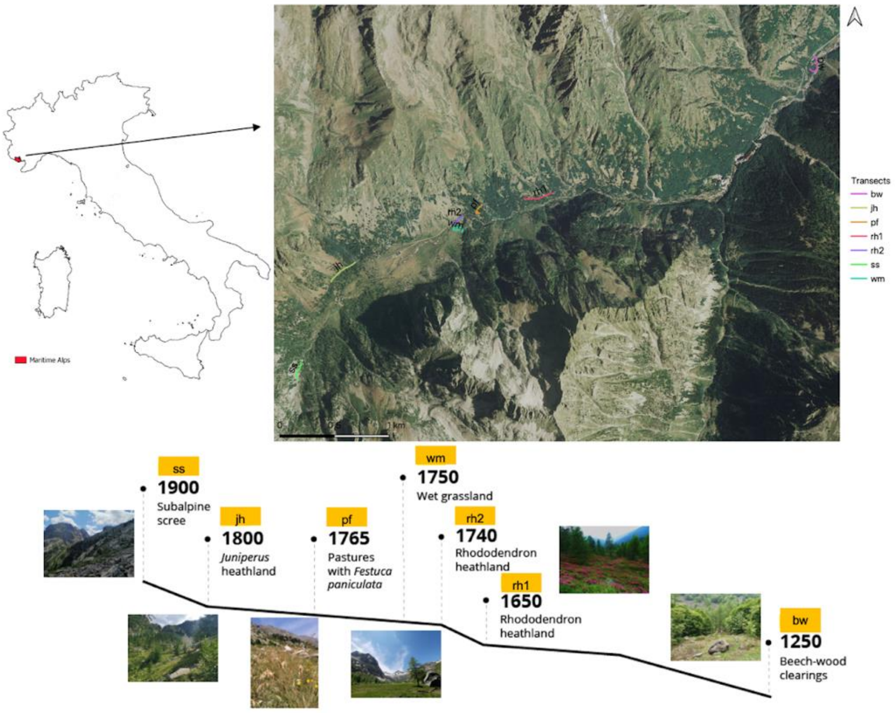

Figure S1. Location and altitudinal gradient of sites surveyed by transects (pollard walk) in the Valasco Valley (SW Italian Alps).

Figure S2. Frequency distribution of the species-by-species number of "gained" or "lost" plots. The median value in red (red line). In each figure, the delta values regarding a) 2009 and 1978 community, b) 2019 and 1978 community, and c) 2019 and 2009 community.

a)

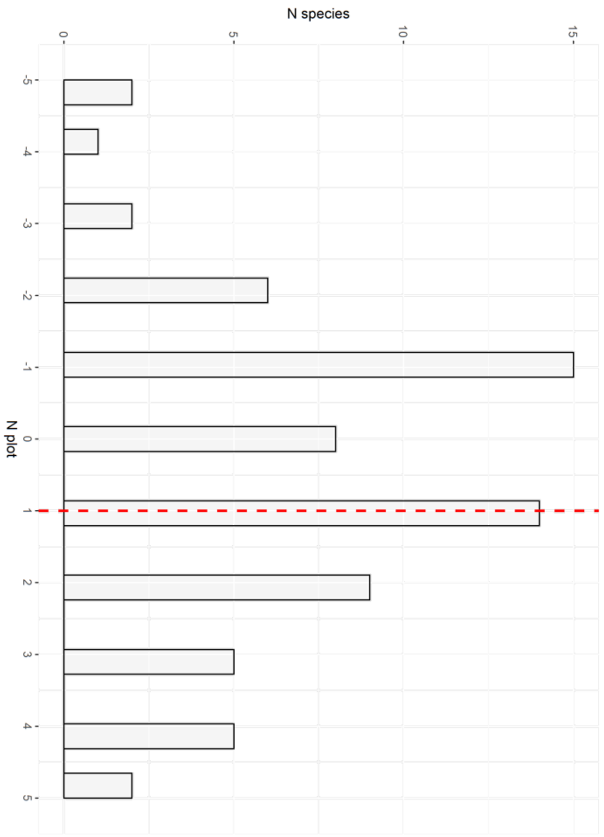

b)

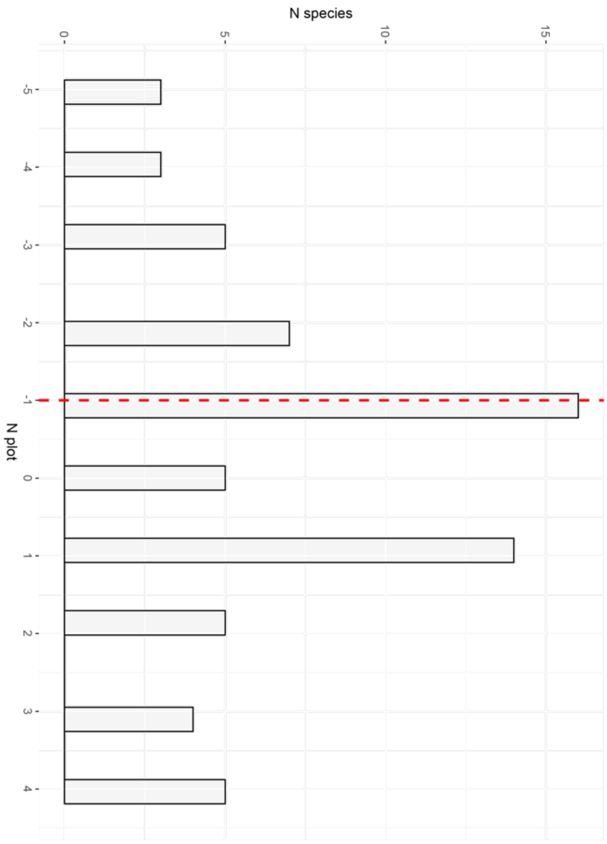

c)

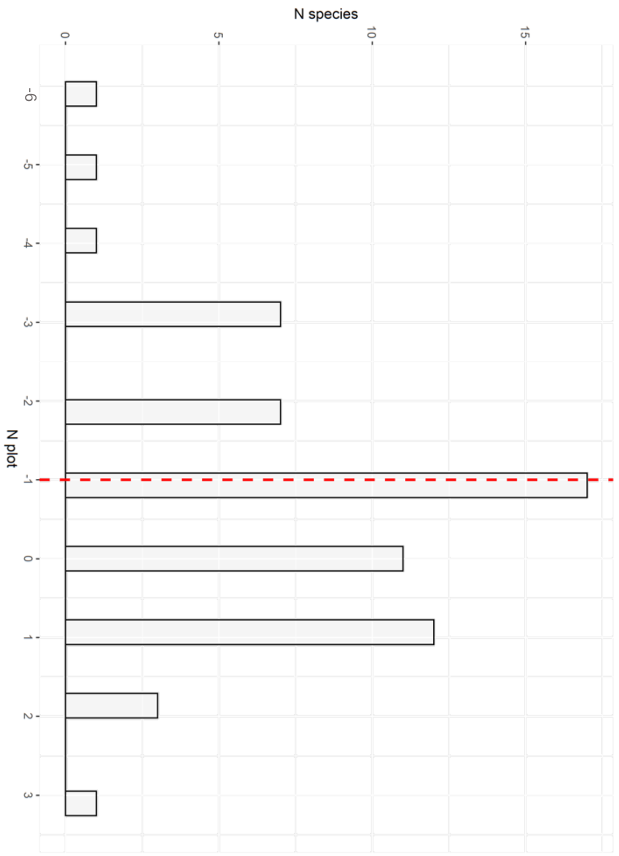

Supplement: Supplementary file 1 [file insects-13-00043-s001.zip › insects-1500183-supplementary.pdf]
